# The relationship between high residential density in student dormitories and anxiety, binge eating and Internet addiction: a study of Chinese college students

**DOI:** 10.1186/s40064-016-3246-6

**Published:** 2016-09-15

**Authors:** Zhuoli Tao, Gao Wu, Zeyuan Wang

**Affiliations:** 1 Department of Medical Humanities, School of Humanities, Southeast University, 87#, Ding Jia Qiao, Nanjing, 210009 China; 2 School of Economics and Management, Nanjing University of Science and Technology, Nanjing, China; 3 School of Biological Science and Medical Engineering, Southeast University, Nanjing, China

**Keywords:** Residential density, Anxiety, Internet addiction, Chinese, Gender difference

## Abstract

**Context:**

Although various studies have indicated that high residential density may affect health and psychological outcomes, to our knowledge, there have been no studies regarding the predictive nature of crowded living conditions on binge eating and the use of the Internet as coping strategies.

**Methods:**

A total of 1048 Chinese college students (540 males and 508 females) were randomly selected and asked to complete a battery of questionnaires that included the Zung’s Self-Rating Anxiety Scale, the Internet Addiction Test, and Rosenbaum’s Self-Control Scale. Binge eating behaviors and compensatory behaviors were also reported, and variables about residential density were measured.

**Results:**

Among female participants, binge eating scores were significantly predicted by anxiety caused by high-density living conditions (*P* = 0.008), and similarly, the frequency of compensatory behaviors was significantly predicted by anxiety caused by high-density living conditions (*P* = 0.000) and self-control (*P* = 0.003). Furthermore, the Internet Addiction Test scores were significantly predicted by the anxiety caused by high -density living conditions (*P* = 0.000) and self-control (*P* = 0.000). Among male participants, not only were the binge eating scores significantly predicted by the anxiety caused by high-density living conditions (*P* = 0.000) and self-control (*P* = 0.000), but the frequency of compensatory behaviors was also significantly predicted by the anxiety caused by high-density living conditions (*P* = 0.000) and self-control (*P* = 0.01). Furthermore, Internet Addiction Test scores were significantly predicted by anxiety caused by high-density living conditions (*P* = 0.000) and self-control (*P* = 0.000). It was further found that for both genders, subjective factors such as self-control, and the anxiety caused by high-density living conditions had a stronger impact on Internet addiction than objective factors, such as the size of the student’s dormitory room. Moreover, self-control was found to act as a moderator in the relationship between anxiety and Internet addiction among male participants.

**Conclusion:**

Binge eating and Internet use could be considered coping strategies for Chinese college students facing high residential density in their dormitories.

## Background

### High-density living conditions and mood

In 1962, Calhoun conducted a series of experiments with rats living in high population densities. At the end of the experiment, the surviving rats presented abnormal psychological outcomes that included asexuality and total withdrawal (Calhoun 1962). Therefore, a number of researchers have paid increasing attention to the relationship between high-density living conditions and psychological outcomes. Studies of adults have revealed that interior residential density is associated with elevated psychological symptoms among adults including mild depression, anxiety and social withdrawal (Evans et al. 1989, 2001).

### Relationship between mood, binge eating and Internet addiction

Studies with humans and animals have shown that stress and the pathological emotional responses to stress are considered as predominant factors in increasing the risk of the subsequent development and persistence of binge eating (Consoli et al. 2009).

There are different theories that attempt to explain the relationship between binge eating and stress, for example, the affect-regulation theory, which postulates that humans engage in binge eating to regulate negative emotional states (Engelberg et al. 2007). The escape theory suggests that individuals who suffer from binge eating want to “escape” from the stimulus environment by narrowing the focus of their attention from more abstract levels to their immediate physical surroundings (e.g., food; Engelberg et al. 2007). This process, called “cognitive narrowing,” serves as an “escape” from distressing self-awareness. A third theory states, that caloric restriction (dieting) interacts with stress in a unique manner that triggers binge eating (Hagan et al. 2003). It posits that binge eating in this model is motivated by reward, not metabolic need (Hagan et al. 2003). This notion supports the central reward opioid system’s contribution to binge eating behavior (Boggiano et al. 2005). Palatable food is thought to enhance dopamine release in brain reward structures including the prefrontal cortex and the nucleus accumbens (Consoli et al. 2009). Furthermore, researchers have found that highly palatable food can trigger binge eating in rats, which suggests that biological processes or pharmacological drivers may be more influential in human binge eating than is currently considered (Hagan et al. 2003).

Internet addiction has also been reported to be a comorbidity condition associated with anxiety disorders (Shapira et al. 2000), substance abuse (Claes et al. 2012), psychosomatic disorders (Claes et al. 2012; Tao 2013), and impulsive control disorders (Mazhari 2012). For example, for many people, the Internet serves as an escape from reality that allows them to cope with the pressures of everyday life (Leung 2004; Chou et al. 1999), as evidenced by the fact that they experience pleasure when communicating online with others (Leung 2004).

### Self-control and environmental demands

It is commonly acknowledged that people use a variety of strategies to cope with chronic environmental demands that tend to become over-generalized once the stressors no longer exist (Evans and Cohen 1987). As a way of coping with crowding, social withdrawal, which includes increased interpersonal distance, reduced eye contact and decreased effort to initiate conversations (Evans et al. 1989), and an increased preference to be left alone (Baum and Valins 1977) have been reported in both labor studies and in home-based studies.

Although the negative effects of environmental stressors are decrease when individuals exhibit high levels of personal control, when people remain in crowded conditions, an essential feature of the experience is that they lose a certain degree of their ability to control what happens to them (Sinha and Nayyar 2000). Persons with a degree of self-control also possess more self-regulatory resources that allow them to control their thoughts, behaviors, and emotions (Barber et al. 2009). Thus, the individuals with high levels of self-control are better able to cope in stressful situations than individuals with low self-control, and the difference in the way the two cope with the situation, i.e., the differential appraisal or interaction, results in different psychological outcomes (Rosenbaum and Ben-Ari 1985).

Self-control is considered an organized set of attributes involved in the self-regulation of, for example, cognition, emotion, and behavior (Barkley 1997; Logue 1988, 1995). The Internet users in this study experienced control over the computer and the Internet. This sense of control can be realized in the human–computer context by a series of commands (Kandell 1998). Self-control has also shown a close relationship with binge eating disorders. One of the key symptoms of binge eating disorders is a sense of lack of control over eating, implying pervasive difficulties in self-control processes (Kittel et al. 2015).

The aim of this study is to examine the relationship between Residential density variables, variables for psychological factors and binge eating, compensatory behaviors, and Internet addiction.

We hypothesize the following:

1. Residential density variables (such as the number of people sharing a room,^1^ the area of the dormitory room,^2^ and the per capita volume^3^) and variables for psychological factors (such as anxiety caused by high-density living conditions and self-control) significantly predict binge eating, compensatory behaviors, and Internet addiction.
2. Self-control can act as a moderator in the relationship between anxiety caused by high-density living conditions and binge eating, compensatory behaviors and Internet addiction.

## Methods

### Design

#### Participants

This survey was approved by the research committee of the School of the Humanities, National Southeast University, China. The survey was administered between February 2011 and October 2011 and involved one university in Nanjing and one university in Chongqing.

A cohort of 1200 Chinese college students randomly received the test battery. The participants were selected from all of the college students in which students take part in an English course (3647 students) in the universities, and one in every three the participants were selected according to their students identification number. Of the 3647 students, 1215 students were selected to receive the survey, and among them, 15 students refused to participation. Of the 1200 test batteries, 88 were not returned and 64 were incomplete and thus excluded, for a response rate of 92.5 %. Of the participants, 508 were females (48.4 % of respondents) and 540 were males (51.6 % of respondents). The mean age of the female students was M = 20.6 years (SD = 1.2) and that of the male students was M = 20.8 years (SD = 1.1). School officials, lecturers and students had previously received a written description of the survey. The students were also informed that participation in the study was voluntary and that they could withdraw at any time. The students responded to the self-administered questionnaires in a classroom during a 20- to 30-min session.

The living conditions were measured by a trained researcher who physically visited one of the dormitory rooms and determined the dimensions using a tape measure. The size of the rooms in Chongqing and Nanjing was standardized by gender. In Chongqing, four female students shared an 19.48 m^2^ by 2.90 m high room, while four male students shared an 18.65 m^2^ by 2.90 m high room. In Nanjing, four to six female or male students shared a 23.64 m^2^ by 3.41 m high room.

### Measures

#### Questionnaires

All the participants completed the questionnaire, which included demographic questions such as the age of the participant and parental education as well as a battery of three tests, namely Zung’s Self-Rating Anxiety Scale, the Internet Addiction Test, and a Self-Control Scale, and questions about binge eating and compensatory behaviors.

The demographic characteristics, which included ethnic group, gender, age, weight, ideal weight and body height were self-reported by the participants.

1. Zung’s Self-Rating Anxiety Scale

This questionnaire (Zung 1971) is a 20-item, 4-point self-reported measurement designed to measure symptoms of anxiety. The scale, which has been translated into Chinese and previously administered in China, demonstrated good validity and reliability (Wu 1990, 1999; Tao and Gao 1994). All 20 items on the survey had been previously adjusted to measure anxiety as perceived to be caused by population-density. For example, the original first item, “Do you feel more nervous and anxious than usual?” was adjusted to read “when there are a number of people in your dormitory room, do you feel more nervous and anxious than usual?” Similar adjustments were made to the remaining 19 items. In the present survey, the Cronbach’s alpha for female and male participants was 0.81.

2. Internet Addiction Test

This questionnaire (Young 1998) is a 20-item, 5-point self-reporting measurement designed to assess the degree of respondents’ Internet use. Higher scores reflect a higher level of problematic Internet use. The questionnaire exhibits adequate reliability and validity (Widyanto and McMurran 2004). In the present survey, the Cronbach’s alpha for female participants was 0.85 and it was 0.89 for male participants.

The Internet Addiction Test was translated into Mandarin Chinese by the first author and was administered to Chinese participants with acceptable validity and reliability (Tao 2013).

3. The Self-Control Schedule

The questionnaire was developed by Rosenbaum (1980) and contains 36 items that enable the participants to rate their level of self control (Sinha and Nayyar 2000).

The SCS has been reported to be a reliable measure, and a number of studies have supported its validity as a measure of self-control behaviors (Rosenbaum 1980). In the present survey, the Cronbach’s alpha was 0.88 for female participants and was 0.82 for male participants.

The SCS was translated into Mandarin Chinese by the first author. The Chinese version was then retranslated into English by an independent translator (an associate professor of English). The retranslated version was found to closely match the original and any differences between the two versions were reconciled.

4. Binge eating and compensatory behaviors

At the beginning of the study, we explained it to participants the symptoms of binge eating as according to the DSM-IV criteria (APA 2000) i.e., recurrent episodes of binge eating in which the amount of consumed food is significantly higher than that consumed by most people in similar conditions. Binge eating is defined as the consumption of a large amount of food (e.g. a large carton of ice cream or a large bag of chocolates) in a short period of time (e.g. 2 h), accompanied by a loss of control over the eating behavior, that is followed by feelings of guilt. There were two questions on the survey that assessed the respondents’ binge eating characteristics. The first question was “Have you engaged in binge eating in the last 3 months?” The response options were “yes” and “no”. The second question was “How frequently have you engaged in binge eating in the last 3 months?” The response options included “one or more times a day”, “two to six times per week”, “once a week or less” and “no binge eating”. The questions have been used in a previous study (Tao 2013).

### Recurrent inappropriate compensatory behaviors

Recurrent inappropriate compensatory behaviors such as (vomiting, excessive exercise and diet pills) are one of the DSM-IV criteria for bulimia nervosa (APA 2000). Two questions were used to determine the respondents’ tendencies to engage in these behaviors. The first question was “Have you engaged in compensatory behaviors (such as self-induced vomiting, excessive exercise, or taking diet pills) to prevent weight gain during the last 3 months?” The respondents answered either “yes” or “no”. The second question was “How frequently have you engaged in these compensatory behaviors to prevent weight gain during the last 3 months?” The response options included “one or more times every day”, “two to six times per week”, “once a week or less” and “no compensatory behaviors”. The question has been used in a study (Tao 2013).

### Statistics

#### Data analysis

Analyses were performed in SPSS 18.0 (SPSS Inc., Chicago, IL, USA), and a multivariable regression was used to identify the significant predictors of binge eating, compensatory behaviors and Internet addiction.

Interaction terms were included in the analyses to determine whether binge eating, compensatory behaviors and anxiety were moderated by self-control. Interaction terms were also included in the analyses to determine whether Internet addiction and anxiety caused by high-density living condition were moderated by self-control.

We also addressed multicollinearity by centering the continuous variables. The VIF scores, which varied from 1.00 to 2.14, suggested that multicollinearity was not a substantive problem in the data, thus allowing for a meaningful interpretation of the results.

Statistical significance was based on two-sided tests evaluated at a 0.05 level of significance.

## Results

According to Table 1, the demographic data and personal characteristics between female and male participants were compared. Multivariable regression was used to identify the predictors of binge eating scores, frequency of compensatory behaviors and Internet addiction. “Age”, “father’s level of education” and “mother’s level of education” and “BMI” served as covariate variables, while binge eating scores, frequency of compensatory behaviors and Internet Addiction Test scores served as the dependent variables. The following variables served as independent variables: the number of people sharing the room, the area of the dormitory room, per capita volume of the dormitory room, anxiety caused by high-density living conditions and self-control. The variables were entered into a block stepwise regression.

**Table 1:** Comparison the female and male participants according their general characteristics

	Female participants	Male participants
	N = 508	N = 540
	M (SE)	M (SE)
Age	20.56 (1.15)	20.79 (1.19)
Height	160.82 (5.81)	171.45 (5.61)
Weight	50.77 (6.20)	61.54 (8.81)
BMI	19.61 (2.02)	20.89 (2.53)

Among female participants, binge eating scores were significantly predicted by anxiety caused by high-density living conditions (β = 0.02, R^2^ change = 0.02, *P* = 0.008), while the frequency of compensatory behaviors was significantly predicted by anxiety caused by high-density living conditions (β = 0.03, R^2^ change = 0.08, *P* = 0.000) and self-control (β = −0.01, R^2^ change = 0.03, *P* = 0.003).

Among male participants, binge eating scores were significantly predicted by anxiety caused by high-density living conditions (β = 0.06, R^2^ change = 0.15, *P* = 0.000) and self-control (β = −0.02, R^2^ change = 0.03, *P* = 0.000). Frequency of compensatory behaviors was significantly predicted by anxiety caused by high-density living conditions (β = 0.05, R^2^ change = 0.25, *P* = 0.000) and self-control (β = −0.01, R^2^ change = 0.02, *P* = 0.01).

Among female participants, Internet Addiction Test scores were significantly predicted by anxiety caused by high-density living conditions (β = 0.60, R^2^ change = 0.14, *P* = 0.000) and self-control (β = −0.21, R^2^ change = 0.08, *P* = 0.000).

Among male participants, Internet Addiction Test scores were significantly predicted by self-control (β = −0.39, R^2^ change = 0.19, *P* = 0.000) anxiety caused by high-density living conditions (β = 0.54, R^2^ change = 0.12, *P* = 0.000). The per capita volume of the dormitory room was not significantly, but shown a trend toward predicting Internet addiction (β = 0.079, R^2^ change = 0.006, *P* = 0.098). The variables of age, father’s level of education and mother’s level of education were controlled. We found that, the interaction between self-control and anxiety caused by high-density living conditions did not significantly predict binge eating scores among either female or male participants (*P* > 0.05).

As Table 2 shows, the variables of age, father’s level of education and mother’s level of education were controlled. Among the male participants, the interaction between self-control and anxiety significantly predicted Internet addiction (β = −0.01, R^2^ change = 0.01, *P* = 0.03). It was further determined that self-control acted as a moderator in the relationship between anxiety and Internet addiction among male participants. However, this finding was not supported among female participants.

**Table 2:** Multiple regression analysis testing for moderator (self control as moderator)

Predictor	Dependent	Beta	*P* value	R^2^ change (%)
Male				
First step	Internet addiction			
Ethnic group		2.47	0.12	
Age		1.08	0.08	
Father education level		1.22	0.32	
Mother education level		−1.79	0.16	
Second step	Internet addiction			
SAS		0.40	0.00**	12
SCS		−0.32	0.00**	12
Third step	Internet addiction			
SAS * SCS		−0.01	0.03*	1
Female				
First step	Internet addiction			
Ethnic group		0.83	0.58	
Age		1.24	0.03*	
Father education level		0.48	0.67	
Mother education level		1.91	0.13	
Second step	Internet addiction			
SAS		0.56	0.00**	14
SCS		−0.17	0.00**	5
Third step	Internet addiction			
SAS * SCS		−0.01	0.53	

**Fig. 1:**
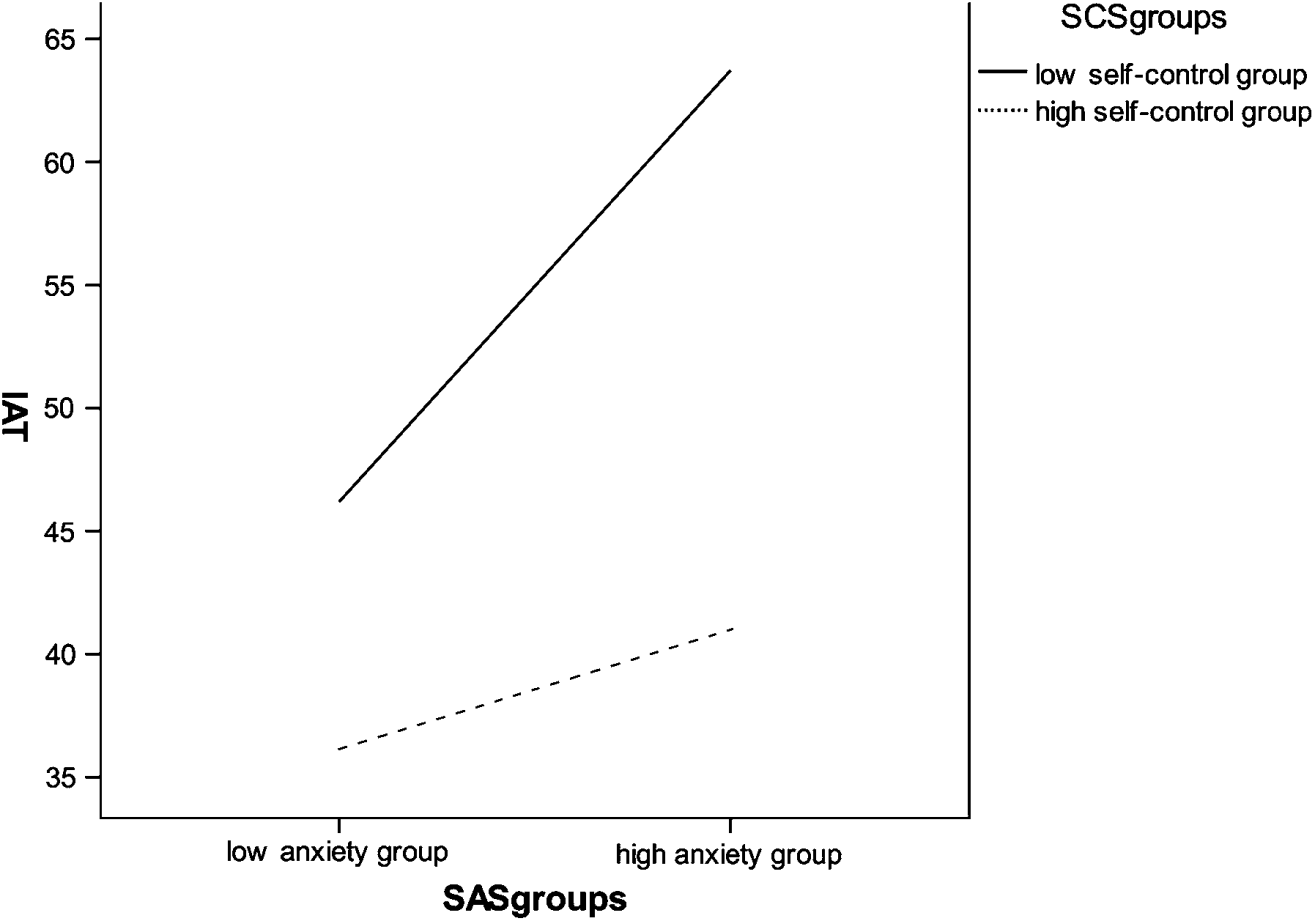
Self-control as moderator of the relationship of anxiety and the Internet addiction among all participants

High anxiety was more strongly associated with higher Internet addiction scores than low anxiety among all participants (Fig. 1), and with relative risk as the degree of self-control decreased. Compared with participants with higher levels of self-control, those with less self-control had a progressively higher likelihood of reporting higher Internet addiction scores.

## Discussion

In our study, we found that the anxiety caused by high-density living conditions could predict binge eating. It partly supported the first hypothesis. Binge eating “can not easily be seen as a direct expression of the concern about shape and weight” (Fairburn and Carter 1997). Emotional deregulation causes maladaptive attempts to regulate mood (e.g. binge eating; Pratt et al. 2001). This emotional deregulation is not necessarily caused by concerns about weight and shape, binge eating can be caused by environment stimulation such as watch video clips including negative emotions, among human participants (Svaldi et al. 2010). The co-occurrence of forced swimming stress and food restriction among mice triggers binge-like behavior (Consoli et al. 2009). A study found that participants with binge eating disorders reported a greater hunger and desire to eating as a result of physical stressor than did obese participants without binge eating disorders, and our results showed that anxiety caused by high-density living conditions can predict the binge eating. The stress stimulating binge eating is not necessarily correlated with weight concern; there could be another reasons that explain it.

Feelings of high anxiety as a result of environmental stimulation could be caused by personality traits, for example low self-control. One of the key symptoms of binge eating disorders is implies pervasive difficulties in self-control processes (Kittel et al. 2015). Our results showed that the self-control could significantly predict binge eating in participants of both genders. This partly supported the results of Davis et al. (2008), who found that the individuals with binge eating disorders had more anxiousness, impulsivity and addictive personalities than normal weight controls. Individuals with binge eating disorders displayed comparatively high levels of expression of anger and attempts to control the expression of anger, as did individuals with bulimic features and healthy controls (Waller 2003).

Low self-control could also be caused by their cognitive and emotional dysfunction. Individuals with binge eating disorders were found to have a dysfunctional use of various emotional regulation strategies (Aldao et al. 2010). Compared to the healthy controls, individuals with binge eating reported difficulties in emotional regulation and emotional awareness (Brockmeyer et al. 2012).

### The relationship between Internet addiction and influencing factors

Self-control and anxiety caused by high-density living conditions can also predict Internet addiction, it also support the first hypothesis.

It was found that for both female and male participants, subjective factors, such as self-control and anxiety caused by high-density living conditions had a stronger impact on Internet addiction than the objective factors, such as per capita volume of one’s dormitory room. In other words, subjective feelings exhibited a stronger influence on Internet addiction than did objective physical factors.

The per capita volume has a trend-level influence on psychological outcome and is a very important environmental factor. To our knowledge, there has been no Chinese research examining the relationship between per capita volume and psychological outcome, but some researchers in China have found that people were not satisfied with their living conditions in some Chinese education institutions because of the high living density. According to Peng’s survey about the relationship between kindergarten environments and children’s behavior in Wuhan (Peng 2010, p. 22), 50 % of the participants were unsatisfied with the ventilation, and 39 % of the participants suggested that this dissatisfaction was caused by too many children being in the kindergarten. According to a survey of Chinese college students in Chengdu, 50 % of the respondents were not satisfied with their dormitories, especially the resident density. As college students become older, they hope for more independent space (Zhang 2007, p. 56). In 2001, the Chinese Ministry of Education released a plan that called for undergraduates’ dormitories to reach 8 m^2^, but at many Chinese universities, the per capita volume for undergraduates can only reach 3 m^2^ (Zhang 2007, p.127).

In the present study, self-control was found to moderator of the relationship between anxiety caused by high-density living conditions and Internet addiction, which supported our second hypothesis and gave credence to the statement that individuals can maintain a sense of well-being when there is an optimal balance between self and environment and that this balance can be improved by altering the self to adapt to the world (Rothbaum et al. 1982).

The results of this study suggest that binge eating and Internet use could be considered “medicines” for the anxiety caused by high residential density in Chinese college students who lived in these dormitories, and moreover, these two behaviors were influenced by an individual’s capacity for self-control. In general we can not radically change our environment, but we can change our cognition regarding stress around us and develop a more rational attitude toward life.

Certain limitations of this study must be acknowledged. One limitation is the reliance on a subjective self-reporting survey. Therefore, to confirm the validity of the results, further studies are needed to replicate our findings.

Similarly, a second limitation is that binge eating and compensatory behaviors were measured using only two questions. Again, although this measure has been previously published (Tao 2013), the results of the present survey must be interpreted with caution.

A third limitation is the cross-sectional, self-report design, which limits our ability to make causal inferences about the relationship between the anxiety caused by high-density living conditions and binge eating, compensatory behaviors, and Internet addiction.

In another survey examining the dieting behavior of Chinese female college students, we found that dieting females who binge and purge with others prefer to perform these actions with their roommates. Because most Chinese college students have roommates, they do not have much privacy (Peng 2010, p. 39). The roommates can thus easily detect binging and purging behaviors, and when the roommates are susceptible to dieting, they may learn these behaviors very quickly. We can not rule out the possibility that some students experience anxiety about the potential negative influence of living in such dormitories.

## Footnotes

1 There were 4–6 people sharing an apartment.

2 The area included a room, bathroom and balcony.

3 The per capita volume of the dormitory room = (the area of the dormitory room) × (room height)/(the number of people living in the room).
